# EEG-based measurement system for monitoring student engagement in learning 4.0

**DOI:** 10.1038/s41598-022-09578-y

**Published:** 2022-04-07

**Authors:** Andrea Apicella, Pasquale Arpaia, Mirco Frosolone, Giovanni Improta, Nicola Moccaldi, Andrea Pollastro

**Affiliations:** 1 Department of Electrical Engineering and Information Technology, University of Napoli Federico II, Naples, Italy; 2 Department of Public Health and Preventive Medicine, University of Naples Federico II, Naples, Italy

**Keywords:** Attention, Electrical and electronic engineering

## Abstract

A wearable system for the personalized EEG-based detection of engagement in learning 4.0 is proposed. In particular, the effectiveness of the proposed solution is assessed by means of the classification accuracy in predicting engagement. The system can be used to make an automated teaching platform adaptable to the user, by managing eventual drops in the cognitive and emotional engagement. The effectiveness of the learning process mainly depends on the engagement level of the learner. In case of distraction, lack of interest or superficial participation, the teaching strategy could be personalized by an automatic modulation of contents and communication strategies. The system is validated by an experimental case study on twenty-one students. The experimental task was to learn how a specific human-machine interface works. Both the cognitive and motor skills of participants were involved. De facto standard stimuli, namely (1) cognitive task (Continuous Performance Test), (2) music background (Music Emotion Recognition—MER database), and (3) social feedback (Hermans and De Houwer database), were employed to guarantee a metrologically founded reference. In within-subject approach, the proposed signal processing pipeline (Filter bank, Common Spatial Pattern, and Support Vector Machine), reaches almost 77% average accuracy, in detecting both cognitive and emotional engagement.

## Introduction

Man’s relationship with knowledge is increasingly mediated by technology. Since the second half of the last century, the *digital era*, namely the period of the pervasive use of information and communication technologies in every area of life, has had a major impact on the human learning^1^. Currently, the ongoing Fourth Industrial Revolution (Industry 4.0) further expands the role of technology in learning processes: automated teaching platforms can adapt in real-time to the user skills and the new generation interfaces allow multi-sensorial interactions with virtual contents^2–4^. In the pedagogical domain, the concept of “Learning 4.0” is emerging and it is not just a marketing gimmick^5^. The 4.0 technologies are strongly impacting on the creation, the conservation, and the transmission of knowledge^6^. In particular, the new immersive eXtended Reality (XR) solutions make possible to achieve *embodied learning* by enhancing the catalytic learning role of bodily activities^7^. Furthermore, wearable transducers and embedded Artificial Intelligence (AI) increase real-time adaptivity in Human-Machine Interaction^8^. In detail, in the Learning 4.0 context, the adaptation between humans and machines is reciprocal: the subject learns to use the human-machine interface, but also the machine adapts to human by learning from her/him^9^.

Traditionally, learning how to use a new technology interface was a once-in-a-lifetime effort conducted at a young age. For many people this has occurred with learning to read and write. Recently, the rapidity of technological evolution has been entailing the need to learn how to use several interfaces. The joy-pad, icon, touch/multi-touch screen, speech and gesture recognition are examples of the evolution of new interfaces (hardware and software components).

More specifically, learning to use an interface is a hard task which requires complex cognitive-motor skills. When human beings learned to use the mouse/touchscreen, as well as when they learned to write, read or speak, their minds learned complex cognitive-body patterns^10,11^. Regarding the human-machine interfaces of older generations, the user was autonomously required to explore the different available resources and learn their use. Currently, the Interfaces 4.0 can adapt in real time to the use, by supporting the learning process^2,3,12^. Proper adaptive strategies could be aimed to improve the learner engagement. Indeed, according to the literature, the effectiveness learning process depends on the engagement level of the learner. In a study on the role of learning engagement in technology-mediated learning^13^ and its effects on learning effectiveness, 212 university students were observed during learning Adobe Photoshop. It was reported how learning materials can negatively affect learning engagement, which in turn reduces the perceived learning effectiveness and satisfaction. The role of e-learning on learning engagement and its effectiveness was evaluated in a study on 181 students reporting higher academic results in case of learning engagement^14^. Therefore, the engagement monitoring is a fundamental aspect allowing the machine to adapt to the user.

In this context, *Engagement* stands for concentrated attention, commitment, and active involvement, in contrast to apathy, lack of interest or superficial participation^15,16^. In the learning context, Newman defines engagement as: “the student’s psychological investment in and effort directed toward learning, under-standing, or mastering the knowledge, skills, or crafts that academic work is intended to promote”^17,18^. Moreover, Frederiks defines the student engagement as a meta-construct including behavioral, emotional, and cognitive engagement^19^.

As concerns the engagement measurability, evaluation grids and self-assessment questionnaires (to be filled out by the observer or by the learner autonomously) are traditionally the most used methods for the behavioral, cognitive, and emotional engagement detection^20^. In recent years, measures based on biosignals are spreading very rapidly. Furthermore, the use of physiological sensors allows the adoption of real-time machine adaptive strategies, by detecting cognitive and emotional engagement. Among the different physiological biosignals, the EEG appears to be one of the most promising technology thanks to its low cost, low invasiveness, and high temporal resolution^21–23^. Moreover, the EEG contains a broader range of information about the mental state of a subject with respect to others biosignals^24^. In^25^ an engagement index was proposed to decide when to use the autopilot and when to switch to the manual control during a fly simulator session. The engagement index was $E=\frac{\beta }{\theta + \alpha }$ where $\alpha ,\beta ,$ and $\theta$ are the EEG frequency bands. This index was used as engagement estimator also in learning contexts^20,26,27^. However, the proposed index does not take into account the different engagement types (i.e., cognitive, emotional and behavioural) proposed by the theories previously reported.

In this study, a method for EEG-based cognitive and emotional engagement detection during learning activities is proposed. High wearability is guaranteed by a low number of dry electrodes. This property allows the cognitive and emotional learning engagement detection in daily life applications. Furthermore, the proposed method can be used also in traditional school contexts. For example, acquiring cognitive and emotional engagement data during the lessons can provide (1) real-time feedbacks to the teacher, for maximizing class engagement, and (2) student engagement trends over the time that can be used for academic program adaptation to individuals or to the whole class.

This work is organized as follows: in “Background” Section, a background on the engagement in the learning context is reported. In “Proposal” Section, the basic ideas and the proposed solution are described. Then, in “Methods” Section, the methods are presented. Finally, the experimental results are discussed in “Experimental Results” Section.

## Background

In general, learning a new interface can be traced back to a classic learning problem. In the constructivism framework, learning consists in the construction of the schemes: units of knowledge, each relating to different aspect of the world, including actions, objects, and abstract concepts^28^. When a subject learns a specific pattern, the *neuroplasticity process* is activated modifying the neural brain structure^29^. Once the process is learned, the brain builds a myelinated axon connection system to automate that. The adjacent neurons fire in unison, and more the experience or operation is repeated, more the synaptic link between neurons becomes strong^30^. The automated use of all mental processes as well as the understanding and use of new technologies occurs through the creation of synaptic pathways^31–33^. For instance, Markham et al.^31^ in their work stated: “Histological examination of the brains of animals exposed to either a complex (‘enriched’) environment or learning paradigm, compared with appropriate controls, has illuminated the nature of experience-induced morphological plasticity in the brain [...] that changes in synapse number and morphology are associated with learning and are stable, in that they persist well beyond the period of exposure to the learning experience.” Kennedy et al.^32^ affirmed: “Learning and memory require the formation of new neural networks in the brain. A key mechanism underlying this process is synaptic plasticity at excitatory synapses, which connect neurons into networks.” During life, humans learn new skills or modify the already learned ones by enriching the existing synaptic pathways. Therefore, the introduction of increasingly innovative technologies requires a continuous brain re-adaptation to new interfaces^34^. This effort is more effective when the learner is engaged. An engaged user actuates learning in an optimal way, avoiding distractions, and increasing the mental performance^35,36^.

In^37^, three different types of engagement are proposed: behavioural, emotional, and cognitive engagements. Behavioral engagement focuses on the observable actions during the learning process^38,39^. Emotional engagement regards the impact of emotions on the cognitive process effectiveness and the effort sustainability for the users^40^. Cognitive engagement refers to the amount of cognitive resources spent by the user in a specific activity^39,41,42^.

Different methods for learning engagement detection are proposed in literature^27^. For the behavioral engagement assessment, observation grids (used to support direct observations or video analysis) were proposed^43,44^. For the cognitive and emotional engagement assessment, self-assessment questionnaires and surveys (compiled autonomously by the user) were developed^45,46^. In recent years alternative engagement assessment methods based on physiological sensors have established: heart-rate variability, galvanic skin response, and EEG^47–49^. Among these biosignal, the most promising for engagement assessment is the EEG. As already described, the learning is based on a neurological changes set, and the EEG presents the possibility of studying these neural modifications^20,50–53^. The EEG system is non-invasive, and provides information on brain activity within milliseconds. Recently low-cost solution appeared on the market (i.e. Emotiv epoc$+$ or Muse^54,55^).

It is now commonly used in many applications^56,57^ including the cognitive and emotion engagement assessment as well as the detection of the underlying elements: emotions recognition and cognitive load activity assessment respectively^58–64^.

To achieve a correct metrological reference of the EEG-based cognitive and emotional engagement constructs, a reproducibility problem arises. From emotional point of view, when eliciting a specific emotion, the same stimulus does not often induce the same emotion in different subjects. The effectiveness of the induction can be verified by means of self-assessment questionnaires or scales. The combined use of standardized stimuli and subject’s self-assessment ratings can be an effective way to build a metrological reference for a reliable EEG-based emotional engagement detection^65^. From the cognitive point of view, when the subject is learning, the working memory identifies the incoming information and the long-term memory constructs and stores new schemes on the basis of the past ones. While the already built schemes decrease in the working memory load, the construction of new schemes entails its increase^24,66^. Therefore, increasing difficulty levels allows to induce different cognitive states; the cognitive engagement level grows up according to the difficulty of the proposed exercise increases^67–70^.

## Proposal

This study proposes an EEG-based cognitive and emotional engagement detection method during a learning task. In this section the Basic ideas, the Architecture, and the adopted Processing framework are outlined.

### Basic ideas

The proposed method is based on the following key concepts:

- *EEG-based subject-adaptative system* In the context of learning 4.0, the adaptability of Intelligent Teaching Systems is improved by means of new input channels (EEG).
- *Cognitive and emotional learning engagement detection* the assessment of student engagement is realized considering both cognitive and emotional aspects, according to the Frederiks theory^19^.
- *Within and cross-subject designs* both the approaches are experimentally validated in order to pursue accuracy maximization or calibration-time minimization, respectively.
- *Domain Adaptation procedure in cross-subject case* a Transfer Component Analysis (TCA)^71^ allows to use knowledge acquired on other subjects to simplify the system calibration on a new subject.
- *Wearable system* an ultralight wireless EEG device with few dry electrodes maximizes the wearability.
- *Multi-factorial metrological reference* the system is calibrated by using (1) standardized strategies for inducing different levels of cognitive load, and (2) a public acoustic stimuli dataset to elicit emotions. Moreover, the metrological reference of emotional engagement was confirmed by statistical analysis on the outputs of self-assessment questionnaires.
- *Narrow EEG frequency intervals* the EEG features resolution is improved by a 12-band Filter-Bank, obtained by sub-dividing the traditional EEG five bands (delta, theta, alpha, beta, and gamma).

### Architecture

**Figure 1:**
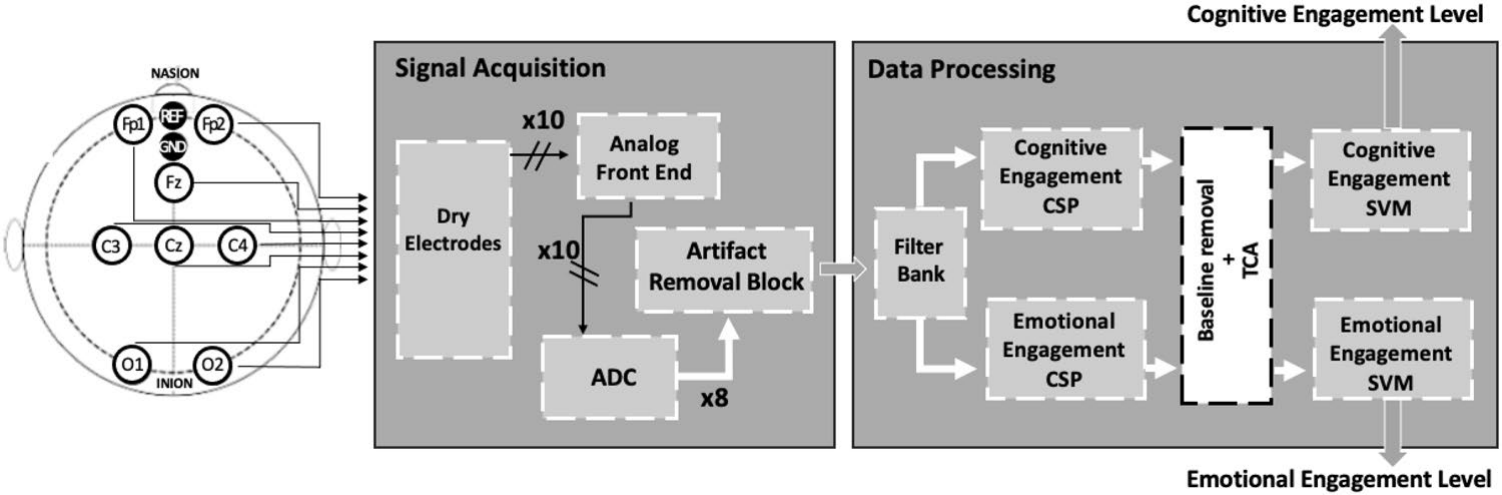
The architecture of the system for engagement assessment; the white box is active only in the cross-subject case (ADC-Analog Digital Converter, CSP-Common Spatial Pattern, TCA-transfer component analysis, and SVM-support vector machine).

The architecture of the proposed system is depicted in Fig. 1. The eight *Active Dry Electrodes* acquire the EEG signals directly from the scalp. Each channel is differential with respect to AFz (REF), and referred to Fpz (GND), according to the 10/20 international system. After transduction, analog signals are conditioned by the *Analog Front End*. Next, they are digitized by the *Analog Digital Converter* (ADC), and submit an *Artifact removal block* performed by an ICA based algorithm. Then, the signals are sent by the wireless Bluetooth transmission to the *Data Processing* stage. Here, the suitable feature are extracted by a 12-component *Filter Bank*. The two *Support Vector Machine* (SVM) classifiers receive the features array from two trained *Common Spatial Pattern* (CSP) algorithms for detecting the Cognitive and the Emotional Engagement respectively. Only in the cross-subject case, a baseline removal followed by a TCA procedure is provided during the training stage of the classifier.

### Processing framework

In this section, (1) the “Feature extraction and selection” section, the (2) “Baseline removal and Domain Adaptation” section, and (3) the “Classification” section are detailed.

#### Feature extraction and selection

In this work, a novel Filter Bank version^57^ is adopted. EEG signals are acquired by an eight channels device with programmable sample rate.

The feature extraction pipeline is based on a filter bank and a Common Spatial Pattern. The filter bank is composed of 12 infinite impulse response (IIR) band-pass Chebyshev type 2 filters with 4 Hz amplitude, equally spaced from 0.5 to 48.5 Hz. The Common Spatial Pattern (CSP)^24^ implements a mathematical data transformation that improves the data separability. The adoption of a pipeline based on Filter-bank and Common Spatial Pattern allows to combine two different goals. Firstly, the EEG frequency spectrum [0.5–48.5] Hz can be investigated, as literature suggests in case of mental state detection^72^. Secondly, by adopting a bank based on 12 filters the resolution of the frequency intervals increases with respect to the five typical bands used in EEG analysis (alpha, beta, delta, gamma, theta). Furthermore, Common Spatial Pattern is widely used in EEG-based motor imagery feature extraction^57,73,74^. Recently, it was demonstrated effective in cognitive^57^ and emotional^75^ mental state detection.

#### Baseline removal and domain adaptation

A cross-subject approach has several advantages with respect to a within-subject one, such as the reduction of time for the initial calibration procedure. Unfortunately, the non-stationarity nature of the EEG signal leads to a greater data variability between subjects. This is a well-known problem in the literature, which makes the cross-subject approach a very challenging task^75^. Currently, the Domain Adaptation methods^76^ are obtaining a great attention from the scientific community. In this work, the Transfer Component Analisys (TCA)^71^ is adopted. TCA is a well-established technique of Domain Adaptation already used in the EEG signal classification literature with promising results^75^.

#### Classification

For the classification stage, Support Vector Machines (SVMs)^77^ are implemented. Considering inputs as points in a vector space, SVM is a binary classifier which discriminates data according to a decision hyperplane. Differently from other hyperplane-based classifiers, an SVM finds the hyperplane maximizing the separation between the classes, i.e. the hyperplane having the largest distance from the *margins* of the classes.

## Methods

In this section the EEG instrumentation, the data acquisition protocol, the data labelling, and the data processing are presented.

### EEG instrumentation

**Figure 2:**
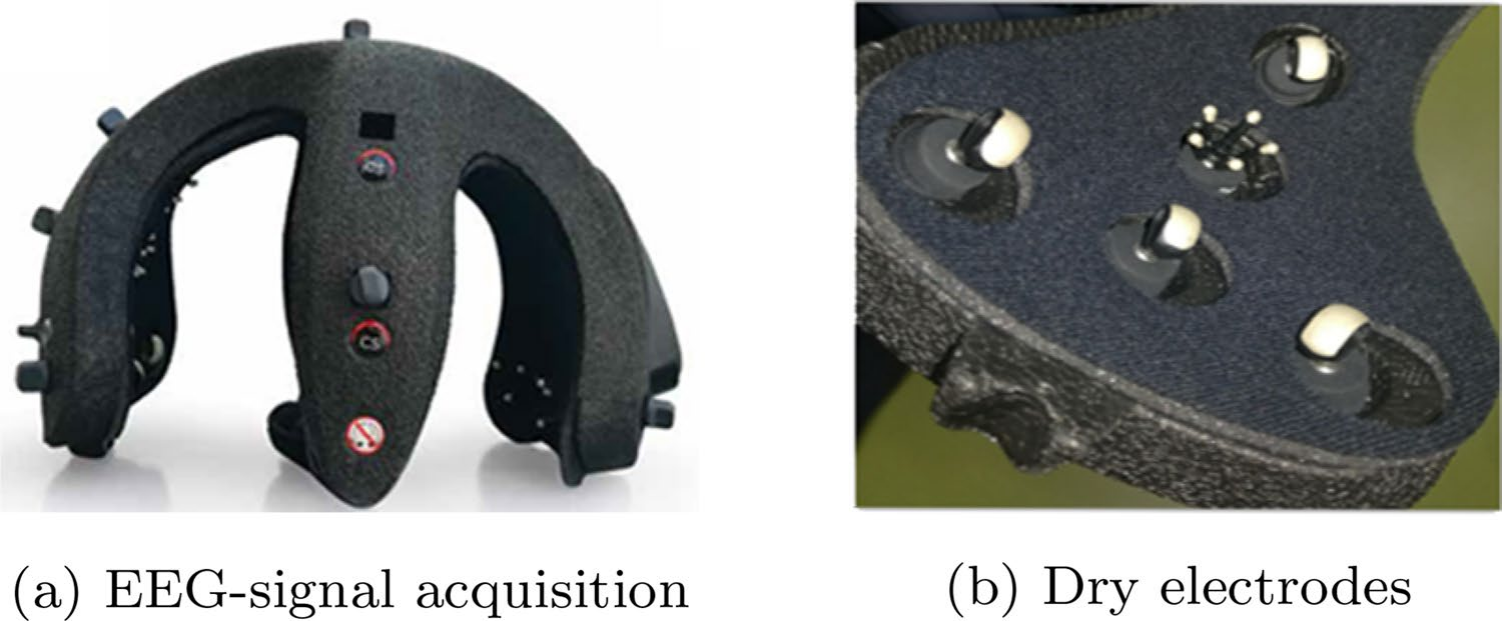
(**a**) EEG-signal acquisition device Helmate8 from abmedica, and (**b**) examples of its dry electrodes^78^.

The AB-Medica Helmate system Class IIA (certified according to the Regulation on medical devices (EU) 2017/745) is used for the EEG signal measurements^78^ (Fig. 2a). The device provides 10 dry electrodes disposed according to the International Positioning System 10/20: Fp1, Fp2, Fz, Cz, C3, C4, O1, O2, AFz (ref), and Fpz (Ground). The signals are differentially acquired with respect to the Fpz electrode and grounded to the AFz electrode. The Electrodes (made of a conductive rubber ending with Ag/AgCl coating) are of three different shapes to minimize the contact impedance in each scalp area (Fig. 2b). The *Helm8 AB-Medica Software Manager*^78^ allows to (1) verify the contact impedance level, and (2) apply several digital filters for a real-time signal visual analysis. The EEG signals are acquired with a 512 Sa/s sampling rate and sent via Bluetooth to a computation device.

### Data acquisition protocol

**Figure 3:**
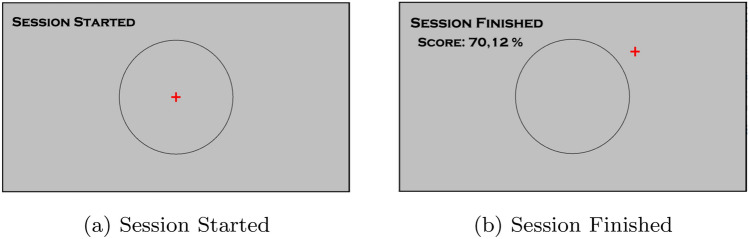
Screenshots from the CPT game. At the beginning of the game (**a**), the cross starts to run away from the center of the black circumference. The user goal is to bring the cross back to the center by using the mouse. At the end of each trial (**b**), the score indicates the percentage of time spent by the cross inside the circumference.

Twenty-one school age subjects (9 males and 13 females, 23.7 ± 4.1 years) participated in the experiment. The experimental sample was extracted from the population of college students in order to soft the impact of age and educational attainment on performance. The ethical committee of the University of Naples Federico II approved the experimental protocol. All methods were performed in accordance with the relevant guidelines and regulations. Before the experiment, each subject read and signed the informed consent. All volunteers have no neurological diseases. Each subject was seated in a comfortable chair at a distance of 1 m from the computer screen. The location was sanitized before and after of each acquisition, as indicated in the COVID-19 academic protocols. Each subject was equipped with a mouse to carry out the experimental test. After putting the EEG-cap on, the contact impedance was assessed to guarantee optimal signal-acquisition conditions. Each subject underwent an experimental session composed of 8 trials. Various stimuli to induce high and low levels of emotive and cognitive engagements were equally distributed among the trials. Continuous Performance Test (CPT)^79^ was used to modulate the cognitive engagement. In particular, a CPT version based on a learning by doing activity on how an interface works was adopted. Whereas, proper background music and social feedback was used to modulate the emotive engagement level. More in detail, the three different stimuli are described as follows:

- *Revised CPT:* a red cross and a black circle on the computer screen were presented to the subject. The red cross tends to run out from the circle on the screen in random directions. The subject was asked to keep the cross inside the circle by using the mouse. For each trial, a different difficulty level was set by the experimenter changing the cross speed. The percentage of the time spent by the red cross inside the black circle with respect to the total time was reported to the subject at the end of the trial (Fig. 3).
- *Background music:* for each trial, a particular emotive engagement level was favored by proper background music. The music tracks were randomly selected from the MER^80^ database where songs are organized according to the 4 quadrants of the emotion Russell’s circumplex model^81^. The songs associated with the Q1 and Q4 quadrants (*cheerful music*) were employed in high emotional engagement trials, Q2 and Q3 for the low ones (*sad music*).
- *Social feedbacks:* during each trial, the experimenters gave proper social feedbacks according to the emotive engagement levels under the experimental protocol. The positive and negative social feedbacks consisted of encouraging and disheartening comments respectively, given to subject on his/her ongoing performance. The positive and negative social feedbacks were administrated using sentences composed of words extracted from a validated database proposed by Hermans and De Houwer^82^ (e.g. *intelligent, game, fast, rule, surprise, applause, good humour, strong, tenacious, skilful, damn, attentive, careless, talented, energetic, music, careless, weak, naive, silly, confused, inexperienced, clumsy, inhibited, great*, etc.). For example, subjects were encouraged and discouraged through comments such as:
  - “*Applause* to you. You did *great*, you achieved a very impressive score in this game. You deserve a round of *applause*. You are a real talent, what a nice surprise.”
  - “*Damn*. You didn’t do very well. You were careless. Shall we try again?”
  The social feedback effectiveness was also improved by the simultaneous music background effects.

A well-founded metrological reference is ensured by two assessment procedures validating the stimuli effectiveness:

- *Performance index:* an empirical threshold was used to confirm that an appropriate CPT stimuli response was given by the participant. The threshold changed according to the trial difficulty level.
- *Self Assessment Manikin questionnaire (SAM):* the emotional engagement level was assessed by a 9-level version of the SAM. The lower emotional engagement level was associated to the SAM score 1, while the greater one to 9.

The experimental session started with the administration of the SAM to get information about the initial emotional condition of the subject. Then, a preliminary CPT training phase to uniform all the participants starting levels was realized. After this preliminary phase, each trial was implemented by a succession of a CPT stage followed by a SAM administration.

### Data labelling

45 s acquisition EEG signals were labeled according to two parameters: (1) high or low emotional engagement, and (2) high or low cognitive engagement. More in detail, regarding the cognitive engagement, the trials were labeled according to the CPT speed^66,83^, since the higher was the speed the more the cognitive engagement increased^24,66^. Vesga et al. correlate directly the cognitive engagement with the cognitive workload^42^. Many studies show how the changes in game difficulty are correlated with cognitive engagement and cognitive load^68–71,84,85^. In^86^, the concept of *desirable difficulties* is presented in terms of “varying the conditions of learning rather than keeping conditions constant and predictable”. This concept is particularly interesting because it connects together the difficulty of the task, the level of involvement and the effectiveness of learning. In this study, the greater difficulty of the task is supposed to induce an increase in the cognitive resources employed by the participant, only if the performances remain compatible. The percentage of the time spent by the red cross inside the black circle with respect to the total time is the performance index used in this study. In detail, for each trial the performance index was analyzed and the subject was assessed as engaged if the final score was within 20% variation with respect to the baseline. Otherwise, the trial was not included in the dataset. The trials having speed lower than 150 pixels/s were labeled as *low*$_c$, whereas *high*$_c$ were assigned to the trials having speed higher than 300 pixels/s.

As concern the emotional engagement, the trials characterized by cheerful/sad music and positive/negative social feedback were labelled as *high*$_e$/*low*$_e$. For each trial, the SAM results (normalized to the initial pre-session values) were consistent with the proposed stimuli. In fact, a one-tailed t-student analysis revealed a 0.02 P-value in the worst case.

### Data processing

An artifact removal stage preceded the feature extraction and the classification stages. Independent Component Analysis (ICA) was used to filter out the artifacts from the EEG signals using the *Runica* module of the EEGLab tool^85^. Then, data were normalized by subtracting their mean and dividing by their standard deviation.

#### Feature extraction

EEG data were divided in epochs of 3 s, overlapping by 1.5 s. Owing to the sampling rate of 512 Sa/s, for each subject 232 epochs of 1536 samples per channel were extracted.

Five different strategies were compared:

1. *Engagement Index:* to make a comparison with the classical literature approach, the engagement index proposed in^25^ was extracted. Although the Engagement Index was not defined for a particular engagement type, given the experimental setup proposed in^25^, it can be assumed compatible with the cognitive engagement proposed in this work.
2. *Butterworth-Principal Component Analysis* (BPCA): data were filtered by a fourth-order bandpass Butterworth filter [0.5–45] Hz; then, relevant features were extracted using Principal Component Analysis (PCA)^87^ selecting the components explaining the 95% of the total variance.
3. *Butterworth-CSP* (BCSP): data were filtered using a fourth-order bandpass Butterworth filter [0.5 - 45] Hz followed by a CSP projection stage; In a binary problem, CSP works by computing the covariance matrices related to the two classes, simultaneously diagonalized such that the eigenvalues of two covariance matrices sum up to 1. Afterwards, a matrix is computed to project the input into a space where the differences between the class variances are maximized. More precisely, in a binary problem, the projected components are sorted by variances in a decreasing or ascending order: the former, when the projection matrix is applied to inputs belonging to the first class, while the latter when inputs belong to the second class^88^.
4. *Filter Bank - CSP* (FBCSP): data were filtered through a 12 IIR bandpass Chebyshev filter type 2 filter bank with a 4 Hz bandwidth equally spaced from 0.5 to 48.5 Hz, followed by a CSP projection stage.
5. *Domain adaptation (TCA)*: only in the cross-subject approach, a baseline removal and a TCA were adopted. In a nutshell, TCA searches for a common latent space between data sampled from two different (but related) data distributions by preserving data properties. More in detail, TCA searches for a data projection $\phi$ that minimizes the *Maximum Mean Discrepancy* (MMD) between the two distributions, that is:
  $$\begin{aligned} \left| \left| \frac{1}{n_S}\sum \limits _{i=1}^{n_S}\phi (\mathbf {x}_{Si})-\frac{1}{n_T}\sum \limits _{i=1}^{n_T}\phi (\mathbf {x}_{Ti})\right| \right| ^2 \end{aligned}$$
  where $n_S$ and $n_T$ are the numbers of points in the first (*source*) and the second (*target*) domain set respectively, while $\mathbf {x}_{S_i}$ and $\mathbf {x}_{T_i}$ are the $i-$th point (epoch) in the two different sets. The data projected in the new latent space are then used as input for the classification pipeline.
6. *Domain adaptation (TCA) with For-subject average removal*: in general, TCA works with only two different domains, differently from a multiple-subject environment, which can lead to a domain composed of several sub-domains generated by the different subjects or sessions. In^75^, TCA was tested by considering for the first domain a subset of samples from $N-1$ subjects, where *N* is the total number of subjects, and with the data of the remaining subject for the other domain. However, this approach does not take into consideration the fact that different subjects may belong to very different domains, leading to poor results. A simple solution consists in subtracting to each subject a baseline signal recorded from the user, for example, in rest condition. However, this last point requires new subject acquisition. Instead, in this work, an average of the signals for each subject is used as baseline, thus avoiding the need for new signal acquisitions.

#### Classification

The output of the classification stage can be “high” or “low” both for cognitive and emotional engagement. Since we are dealing with a binary classification problem, the theoretical chance level for prediction is 50%. For each feature selection strategy shown in the previous subsection, several classifiers were compared with the adopted SVM: Linear Discriminant Analysis (LDA)^89^, *k*-Nearest Neighbour (*k*-NN)^89^, shallow Artificial Neural Networks (ANN), Deep Neural Networks (DNN)^90^, and Convolutional Neural Networks (CNN)^91,92^. LDA searches for a linear projection of the data in a lower dimensional space, while keeping preserved the discriminatory information between the data classes. *k*-NN is a model that, given a set *P* of non-labelled points to classify, a distance measure *d* (such as the Euclidean distance), a positive integer *k*, and a set *D* of labelled points, assigns to each point $p \in P$ the most frequent class, according to the measure *d*, among its *k* neighbours in *D*. ANN is a model consisting of a set of basic elements (called *neurons*), arranged in several full-connected *layers*. Each neuron computes the linear combination of its inputs, that is subsequently given as input of an *activation function*. The number of neurons, the number of layers and the activation functions are a priori hyperparameters, while the coefficients of each linear combination are learned during a training stage. According to the number of layers, in this work ANNs are referred as *shallow* when they are made by a single layer, otherwise they are referred as *deep* (DNN). CNNs are deep networks inspired by the functioning of the visual cortex of the brain in processing and recognizing images. Differently from classical deep neural networks, CNNs extract features from the input using the mathematical convolution operator.

Each combination of feature selection strategies and classifiers were used on both emotional and cognitive engagement.

**Table 1:** Classifier optimized hyperparameters and variation ranges.

Classifier	Optimized Hyperparameter	Variation Range
*k*-Nearest neighbour (*k*-NN)	Algorithms	{Ball tree, KD Tree, Brute force}
	Distance Weight	{equal, inverse}
	Num Neighbors	[1, 10], step: 1
Support vector machine (SVM)	C Regularization	{0.01, 0.1, 1, 5, 10}
	Kernel Function	{radial basis, polynomial}
	Polynomial Order	{2, 3}
Linear discriminant analysis (LDA)	Solver	{Singular value decomposition, least squares}
	Shrinkage	{None, Ledoit-Wolf lemma}
shallow artificial neural network (ANN)	Activation function	{ReLU, sigmoid}
	Number of neurons	[5, 50], step: 25
	Number of layers	{1}
	Learning rate	{0.001, 0.01}
Deep neural network (DNN)	Activation function	{ReLU, sigmoid}
	Number of neurons per layer	[5, 100], step: 25
	Number of layers	{2,3,4}
	Learning rate	{0.00001, 0.0001, 0.001}
Convolutional neural network (CNN)	Activation function	{ReLU, sigmoid}
	Number of layers	{1,2,3}
	Number of filters	{13,32,64}
	Kernel size	{3, 5}
	Stride	{1, 2}
	Dense layer size	{50, 100}
	Learning rate	{0.001, 0.01}

The best model was selected by a *stratified leave-2-trials out* technique in order to maintain a balancing among the classes in each fold. A *Grid search* strategy was adopted as approach for hyperparameters tuning for each classifier (Table 1).

## Experimental results

In this section, the experimental results obtained in within- and cross-subject cases are reported.

### Within-subjects

Firstly, to make a comparison with the classical literature approach, the engagement index proposed in^25^ was used as feature for a classification of the cognitive engagement. Unfortunately, as highlighted by the results reported in Table 2, accuracy performances were not optimal. In fact, this feature is mainly used in non-predictive applications (e.g.,^27^).

**Table 2:** Within-subject experimental results. Classification accuracies using the *Engagement Index*^25^ for cognitive engagement classifications are reported. CNN classifier is not applicable since the *Engagament Index* consists in a single feature.

Method	Cognitive engagement
SVM	54.8 ± 4.9
k-NN	53.7 ± 5.7
ANN	53.1 ± 5.4
LDA	50.7 ± 6.2
DNN	53.4 ± 3.8

**Figure 4:**
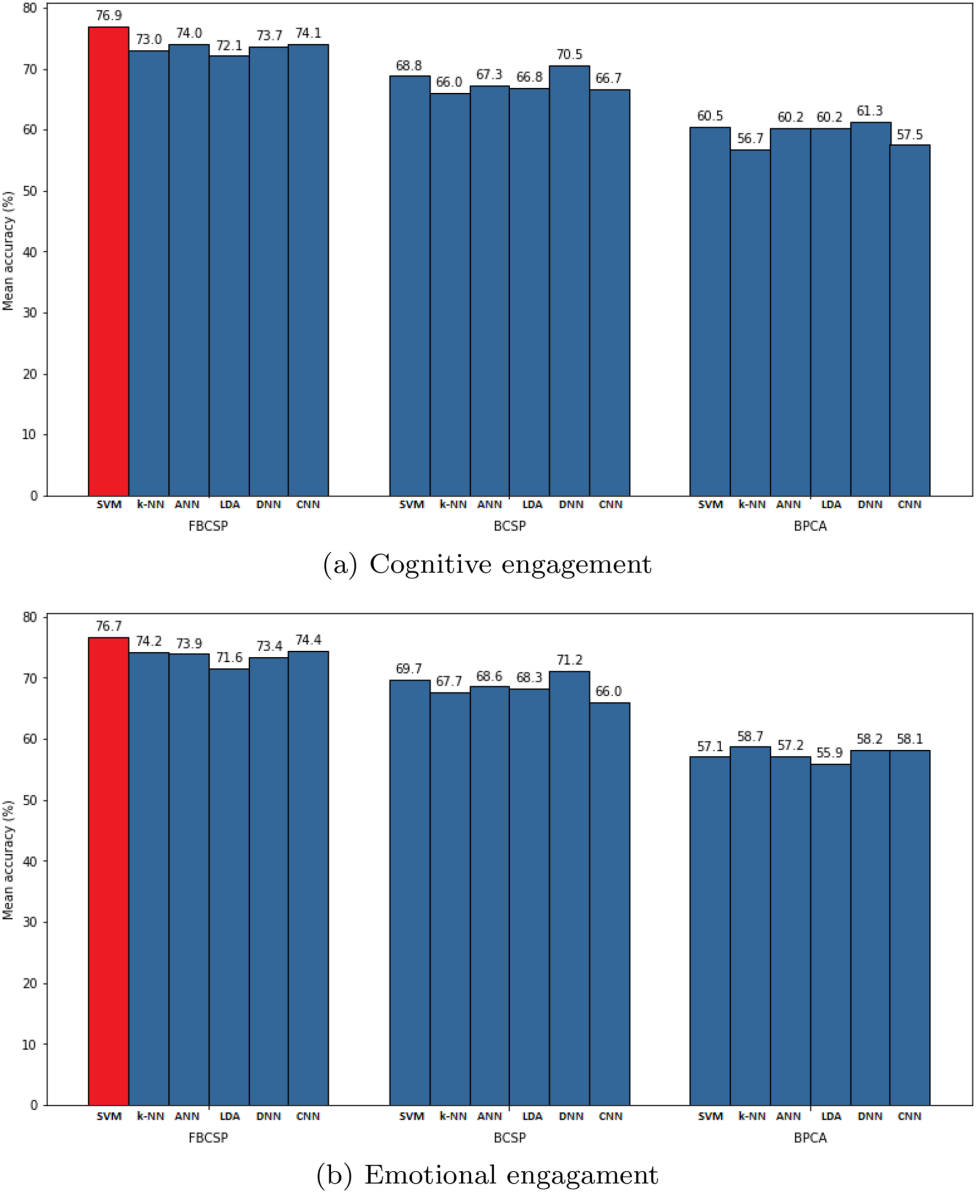
Within-subject performances of the compared processing techniques SVM, *k*-NN, ANN, LDA, DNN and CNN in (**a**) cognitive engagement and (**b**) emotional engagement detection. Each bar describes the average accuracy over all the subjects.

Instead, the best results both on cognitive and emotional engagements (Fig. 4) were achieved using features extracted by Filter-Bank and CSP.

**Figure 5:**
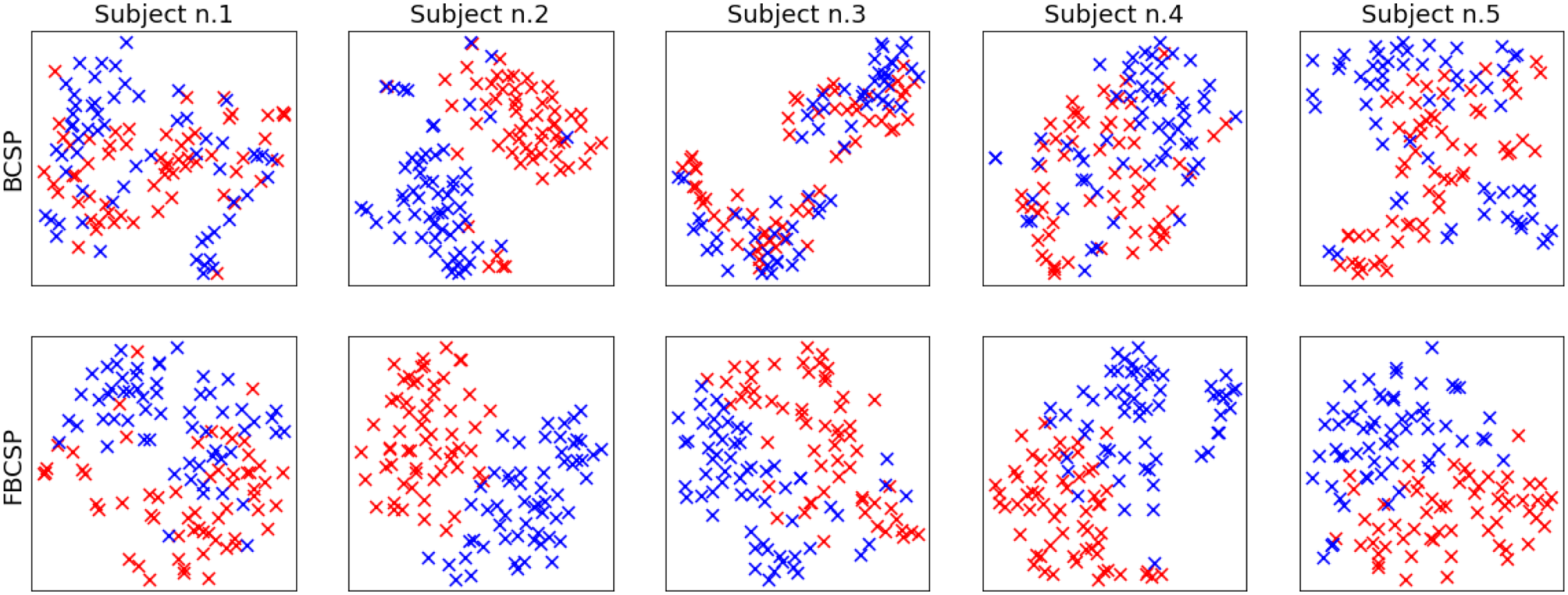
Filter Bank impact on the class (red and blue points) separability. t-SNE-based features plot of five subjects randomly sampled (first row: without Filter Bank; second row: with Filter Bank).

Quantitative results related to the use of Filter Bank and CSP for each classifier can be observed in Table 3: among the different classifiers, SVM stands out with a better performance than the other ones, reaching its best mean accuracies of $76.9 \pm 10.2$ on cognitive engagement classification and of $76.7 \pm 10.0$ on emotional engagement. Results are computed as the average accuracy over all the subjects. Current results suggest that SVMs can be optimal to address the proposed classification problem. A possible explanation is that the kernel spaces induced by the Support Vector Machines resulted particularly suitable for the acquired data size in junction with the features transformation adopted (FBCSP). As shown in Fig. 5, where the Filter Bank effects are represented using t-SNE, FBCSP improves the data separability between the classes and simplifies the classification problem. Therefore, the task can be dealt with a classifier having a low number of parameters identifiable even from datasets not necessarily large.

**Table 3:** Within-subject experimental results. Accuracies are reported on data preprocessed using Filter Bank and CSP for cognitive engagement and emotional engagement classifications.

Method	Cognitive engagement (proposed)	Emotional engagement (proposed)
SVM	**76.9** ± **10.2**	**76.7** ± **10.0**
k-NN	73.0 ± 9.7	74.2 ± 10.3
ANN	74.0 ± 9.2	73.9 ± 9.1
LDA	72.1 ± 11.4	71.6 ± 9.3
DNN	73.7 ± 8.9	73.4 ± 9.6
CNN	74.1 ± 10.1	74.4 ± 9.4

The best performance average values are highlighted in bold.

The results reported in Fig. 2b show that the Filter Bank improves the classification performance by a significant proportion. This can be due to the use of several sub-bands which highlight the signal main characteristics, allowing the CSP computation to project the subject data in a more discriminative common space. In Fig. 5, BCSP and FBCSP are compared through t-SNE^93^ on the subjects data transformed using the two different methods. The figure shows that, for several subjects, CSP applied after FB projects the data in a space where they are easily separable with respect to the BCSP case.

### Cross-subject approach

**Figure 6:**
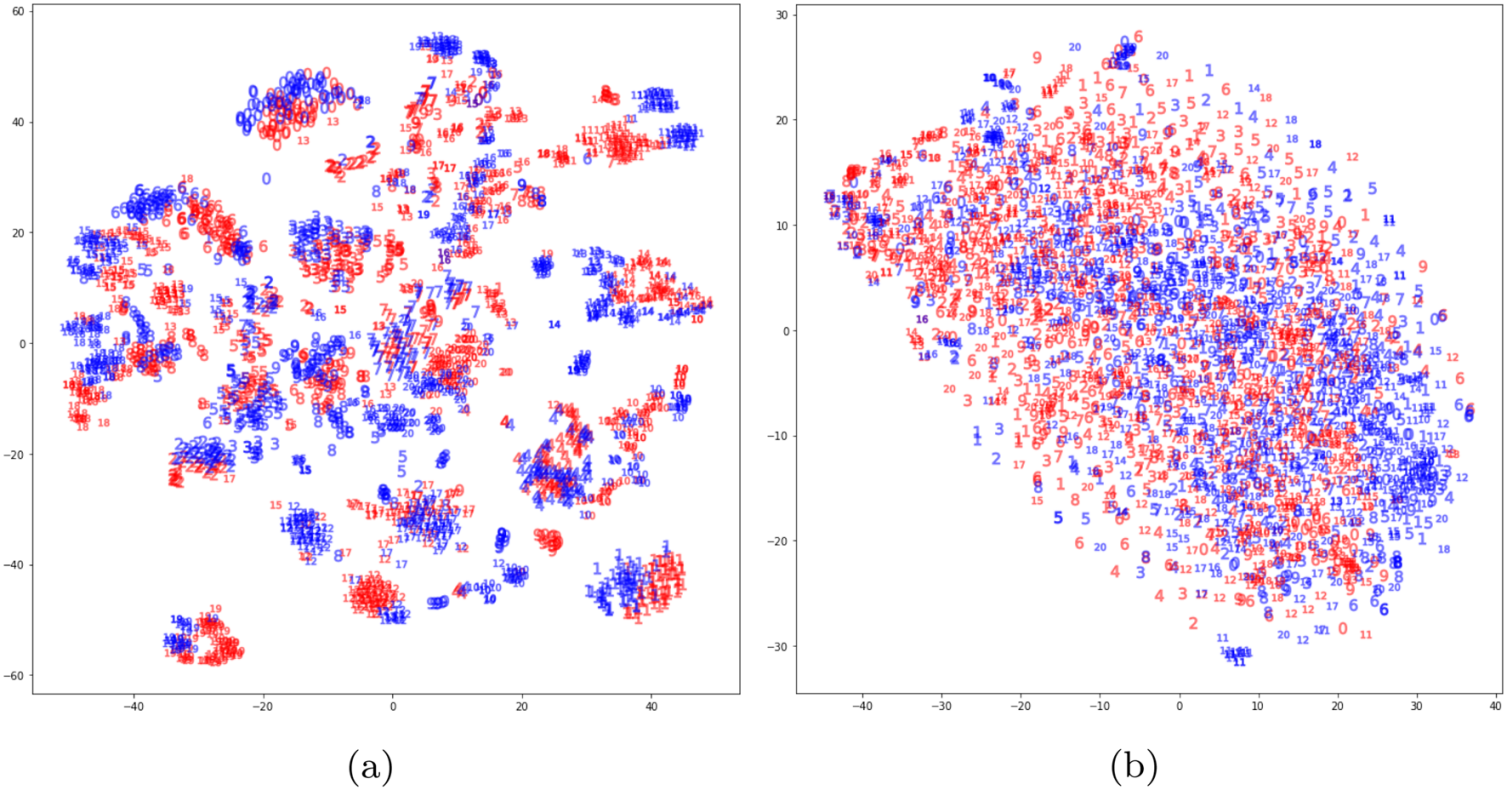
A comparison using t-SNE of the FBCSP data first (**a**) and after (**b**) removing the average value of each subject, in the cross-subject approach. The colors (red and blue) correspond to the two classes, the numbers identify the individuals.

A t-SNE plot of the data first and after removing the average value of each subject is shown in Fig. 6. The data without for-subject average removal (Fig. 6a) are disposed in several clusters over the t-SNE space, exhibiting a fragmentation tendency. Instead, after the for-subject average removal (Fig. 6b), the data result more homogeneous, enhancing the model generalizability. A comparison using TCA with and without the for-subject average removal is made and the resulting performances are reported in Table 4. The results show that removing the for-subject average from each subject boosts the performance with respect to using TCA alone (more than 3% of improvement in almost all classifiers, especially in Cognitive Engagement case).

**Table 4:** Cross-subject experimental results using FBCSP followed by TCA. Accuracies are reported with and without for-subject average removal for cognitive engagement and emotional engagement detection.

Method	With for-subject average removal		Without for-subject average removal	
	Cognitive engagement	Emotional engagement	Cognitive engagement	Emotional engagement
SVM	**72.8** ± **10.9**	**66.2** ± **13.8**	64.0 ± 10.8	61.7 ± 10.2
k-NN	69.6 ± 11.2	61.9 ± 9.1	57.1 ± 8.9	56.9 ± 10.6
ANN	72.6 ± 11.8	65.7 ± 14.1	69.7 ± 12.3	65.8 ± 14.8
LDA	69.5 ± 12.2	65.3 ± 13.8	69.6 ± 12.9	64.6 ± 13.2
DNN	72.1 ± 11.6	66.0 ± 14.0	70.4 ± 13.4	65.8 ± 14.6
CNN	69.9 ± 10.8	64.5 ± 11.6	65.9 ± 12.8	63.8 ± 14.5

The best performance values are highlighted in bold.

## Conclusion

In this work, a wearable system for personalized EEG-based cognitive and emotional engagement detection is proposed. The system can be used in the context of Learning 4.0 as a new input channel of an adaptive automated teaching platform to improve the learning effectiveness. The wearability is guaranteed by a wireless cap with dry electrodes and 8 data acquisition channels.

The system is validated on students during a training stage involving cognitive and motor skills and aimed to learn how to use a human-machine interface. Standard stimuli, performance indicator, and self assessment questionnaires were employed to guarantee a well founded metrologically reference. The proposed method, based on Filter Bank, CSP and SVM, experimentally showed the best performance. In particular, in the cross-subject case, an average accuracy of 72.8% and 66.2% was reached for the cognitive engagement and emotional engagement respectively by using TCA and for-subject average removal. Instead, in the within-subject case, an accuracy of 76.9% and 76.7% was reached for the cognitive engagement and emotional engagement, respectively. This study was conducted in laboratory, therefore a prototype demonstration in operational environment still lacks. In future works, the proposed solution will be tested in real educational situations (e.g. a real lesson) and validated by means of standardized engagement assessment procedures (e.g. self-reports).

## Figures and Tables

**Figure 7:**
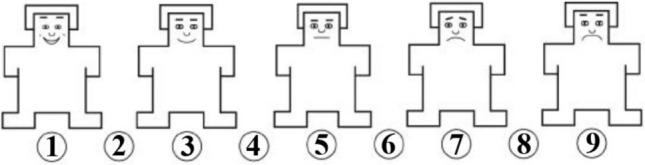
The Self Assessment Manikin. Scale for Valence Assessment.

## Appendix

The Self-Assessment Manikin (SAM) is an iconic assessment questionnaire that directly measures the valence, arousal, and dominance associated with a person’s emotional response^94^. In Fig. 7, the 9-Point version for valence assessment is reported. Specifically, a single-item scale measures valence/pleasure of the response from positive to negative. SAM is freely available questionnaire and it is very intuitive. It requires little explanation and participants generally have no difficulty in completing it. SAM resulted reliable after comparison analysis with other standard assessment tools (i.e., Semantic Differential Scale^95^). It was also shown to be employable in different cultural contexts and with participants of different ages^96^.
